# Home based exercise to improve turning and mobility performance among community dwelling older adults: protocol for a randomized controlled trial

**DOI:** 10.1186/1471-2318-14-100

**Published:** 2014-09-08

**Authors:** Ashari Asmidawati, Tengku Aizan Hamid, Rizal M Hussain, Keith D Hill

**Affiliations:** 1 School of Physiotherapy and Exercise Science, Faculty of Health Sciences, Curtin University, Perth, Western Australia 6845, Australia; 2 Institute of Gerontology, Universiti Putra Malaysia, Serdang, Selangor, Malaysia; 3 Health Promotion Division, National Ageing Research Institute, The Royal Melbourne Hospital, PO Box 2127, Parkville, Melbourne, Victoria 3050, Australia

## Abstract

**Background:**

Turning is a common activity for older people, and is one of the activities commonly associated with falls during walking. Falls that occur while walking and turning have also been associated with an increased risk of hip fracture in older people. Despite the importance of stability during turning, there has been little focus on identifying this impairment in at risk older people, or in evaluating interventions aiming to improve this outcome. This study will evaluate the effectiveness of a 16 week tailored home based exercise program in older adults aged (50 years and above) who were identified as having unsteadiness during turning.

**Methods/Design:**

A single blind randomized controlled trial will be conducted, with assessors blind to group allocation. Study participants will be aged 50 years and above, living in the community and have been identified as having impaired turning ability [outside of age and gender normal limits on the Step Quick Turn (180 degree turn) task on the Neurocom® Balance Master with long plate]. After a comprehensive baseline assessment, those classified as having balance impairment while turning will be randomized to intervention or control group. The intervention group will receive a 16 week individualized balance and strength home exercise program, based on the Otago Exercise Program with additional exercises focused on improving turning ability. Intervention group will attend four visit to the assessment centre over 16 weeks period, for provision, monitoring, modification of the exercise and encourage ongoing participation. Participants in the control group will continue with their usual activities. All participants will be re-assessed on completion of the 16 week program. Primary outcome measures will be the Step Quick Turn Test and Timed-Up and Go test. Secondary outcomes will include other clinical measures of balance, psychological aspects of falls, incidence of falls and falls risk factors.

**Discussion:**

Results of this study will provide useful information for clinicians on the types of exercises to improve turning ability in older people with increased falls risk and the effectiveness of these exercises in improving outcomes.

**Trial Registration:**

ACTRN12613000855729.

## Background

Successful ambulation in one’s daily activities is dependent on the ability to maintain balance during navigation. Balance is defined as the “harmonious and contextually appropriate interplay of stability and mobility of the body with respect to its Base of Support” [1]. Impairments in balance, gait and lower limb strength are important factors associated with reduced mobility and dependency in activities of daily living among older people [2]. Balance performance results from a complex interaction between sensory and musculoskeletal systems requiring constant adjustment of muscle activity and joint position sense and other sensory information to retain the centre of mass of the body over the base of support [3]. In addition, disorders of motor output such as impaired (efferent) reaction time, reduced muscle strength and other factors such as pain can impair balance control [4]. Furthermore, impaired balance is one of the strongest risk factors for falls [5,6].

Turning is a common manoeuvre for older people, with one study reporting 20% of steps during indoor daily activities involving turning [7]. In a study evaluating turning in four activities, including walking through a cafeteria, through a convenience store, and from a specific office to a car in the parking lot, between 8-50% of steps involved a turning manoeuvre [8], highlighting the importance of turning in daily ambulation. Furthermore, turning has been shown to be a more challenging manoeuvre compared to straight-line walking [9]. Older people with balance impairment and history of falls have been shown to have difficulties during turning [10,11]. Falls during turning manoeuvres increase the risk of hips fractures eight times compared to falls during walking [12], and have also been found to be associated with greater risk of slip-related falls [13].

Exercise has been one of the most frequently investigated interventions to reduce falls among older people living in the community [14]. Meta-analyses have shown that exercise programs with a moderate to strong challenge to balance are most likely to be effective in improving functional and mobility performance among older adults, and to reduce risk of falling [14]. However, to date few studies have investigated turning difficulties in older people, and none have investigated the effect of an exercise program which includes specific exercises to improve turning ability in older people with identified turning impairment.

Therefore, the primary aim of the present study is to conduct a randomised controlled trial to evaluate the effectiveness of an individualized home based exercise program in improving turning and common balance impairments among community dwelling Malaysians aged 50 years identified as having impaired turning ability.

## Methods/Design

**Figure 1:**
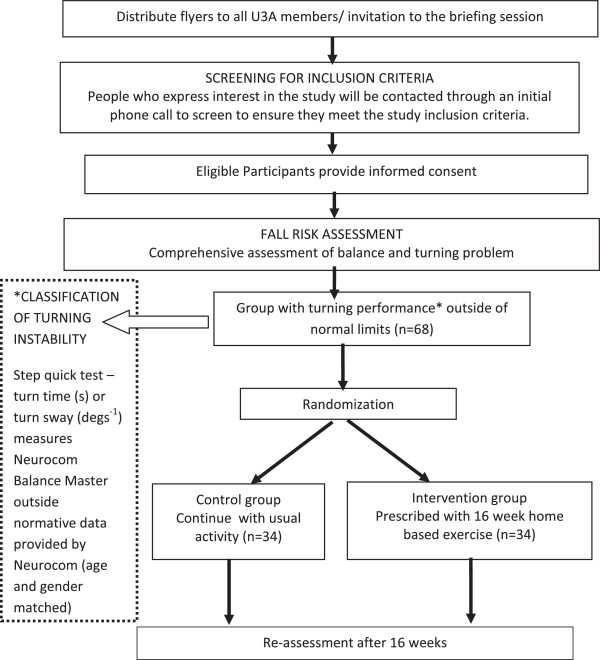
Flowchart of study.

This study is a single blind randomized controlled trial, with the assessors blind to group allocation. The CONSORT statement has been used as a framework for development of methodology for this study [15]. A 16 weeks home based exercise program with a focus on exercises aiming to improve turning ability, as well as other identified balance impairments will be used for the intervention group, while the control group will maintain their usual activities. A flow diagram of the study protocol is illustrated in Figure 1.

### Participants

Participants will be recruited from the membership of the University of Third Age (U3A) of Malaysia, on a voluntary basis. The age of 50 years and above will be used in this study as a criterion for defining “older people” (instead of the more commonly used age of 60 years and above), due to the lower life expectancy in Malaysia (72 years for males and 78 years for females [16]) compared to Western countries. Recruitment of participants will be through distribution of flyers to all U3A members.

Potential participants will be screened on a single occasion prior the baseline assessment to ensure they meet the study inclusion criteria. Participants will be included in the trial if they meet all of the inclusion criteria: i) identified having unsteadiness during turning Neurocom® Balance Master with long plate, and if performance is outside of normal limits (based on normative data based age and gender limits in the Neurocom® system); ii) community-dwelling; iii) aged 50 years and over; iv) able to tolerate standing and walking independently for at least six minutes and community ambulant (able to walk independently outdoors without support or not needing more than a single point stick; v) no other major neurological history (e.g. stroke with unilateral or bilateral paresis, multiple sclerosis) or orthopaedic history that impacts on functional mobility.

Participants will be excluded if they: i) have severe clinical or musculoskeletal impairments (eg; previous fractures) affecting mobility; ii) have visual or auditory impairments which cannot be corrected; iii) have a past history of stroke, Parkinson’s disease, cardiac problems, or transient ischemic attacks; iv) are aged less than 50 years old; v) use a walking frame, crutches or other bilateral support gait aids for walking in the community; vi) are not community ambulant (not able to walk outdoors away from home independently); and vii) are institutionalized (living in residential care).

### Measures and procedures

Participants will undergo a comprehensive assessment including questionnaires consisting of socio-demographic, health and medical conditions, assessment of falls risk, psychological aspects associated with falls, level of physical activity as well as balance and mobility assessment using clinical and laboratory (force platform) tests. Questionnaires will be interspersed between the physical performance tests, and participants will be able to rest between tests if required. A summary of the screening and assessment measures to be used is shown in Table 1.

**Table 1:** Summary of the screening and assessment measures during baseline and after 16 weeks intervention program

**Screening tests**	**Baseline**	**Follow-up (16 weeks)**
Questions: age; standing & walking; independent living; independent community ambulation with no more than single point stick	√	
Cognition (Elderly Cognitive Assessment Questionaire-ECAQ).	√	
Socio-demographic information	√	
Health conditions & medication use	√	
History of falls	√ (past 12 months)	Will be asked at each phone call follow up (preceding 3 weeks) and at 16 weeks (falls diary)
**Assessment – questionnaires/tests**		
Falls efficacy (fear of falling) – FES-I	√	√
Depression– Geriatric Depression Scale 15 items.	√	√
Visual contrast sensitivity (Melbourne Edge Test- MET)	√	
Activity level (Human Activity Profile Adjusted Activity Score - HAP-AAS)	√	√
Falls risk (FROP-Com risk assessment tool)	√	√
Exercise adherence (exercise diary)		√ (intervention group) daily exercise diary
**Assessments – clinical (balance/mobility)**		
Timed up and Go (single task)	√	√
Timed Up and Go (dual task)	√	√
Step Test (ST)	√	√
Functional Reach Test (FRT)	√	√
Five Times sit to stand Test (FTSS)	√	√
**Assessments – force platform**		
Modified Clinical Test of Sensory Interaction Balances (mCTSIB)	√	√
Limits of Stability (LOS)	√	√
Sit to Stand (STS)	√	√
Walk across (WA)	√	
Step quick turn (SQT)	√	√

All of the measures being used in this study have been shown to have moderate to high reliability and validity in a variety of older and clinical populations [17-20]. Assessments will be conducted by a trained assessor blind to group allocation.

Measures of fall risk and fall risk factors;

i. A retrospective recall about any falls in the preceding 12 months. The information regarding history of fall such as date, location, activity at the time of fall, obstacles involved in the fall, any warning sign or symptoms, type of injuries and medical attention sought after fall will be retrieved for each of fall event (details for up of 4 falls will be retrieved).

ii. Falls Risk assessment for Older People in the Community (FROP-Com) [19], a detailed assessment of 13 falls risk factors. Each domain of the assessment describes an evidence based fall risk factor among community dwelling older adults, and each domain is scored to reflect graded risk (nil, mild, moderate, severe). The FROP-Com has been shown to have good retest reliability (ICC for intra-rater and inter-tester reliability were 0.93 and 0.81 respectively) [21] and moderate accuracy predicting falls (sensitivity 71% and specificity 56%) [21]. A total score between 0 and 60 will be obtained, where higher scores indicate greater level of risk of falling.

iii. Activity level will be monitored in both groups at baseline and 16 weeks using the Human Activity Profile (HAP) [22]. The Adjusted Activity Score (AAS) will be used, calculated by subtracting the number of items listed as “stopped doing” from the highest numbered item listed as “still doing”. This will enable identification of any unanticipated change in activity/exercise level in the control group during the 16 week trial; as well as monitor any change in activity level by the intervention group.

iv. Psychological aspects related to falls will be measured using Falls Efficacy Scale-International (FES-I) short version-7 items [23] to assess the level of fear of falling. Level of depression will be measured using the 15 item Geriatric Depression Scale [24].

v. Visual contrast sensitivity will be evaluated using the Melbourne Edge Test (MET) [25]. The correct identification of the orientation of the edges on the circular patches provides a measure of contrast sensitivity in decibel units, where dB = −10log_10_ contrast.

Clinical balance and mobility measures to be administered at baseline will include:

i. Timed Up and Go Test (TUG) [26] will be used to measure dynamic balance and mobility. This assessment will be carried out by using a chair with armrests (seat height approximately 45 cm with armrest height 63 cm) and stop watch. The participant will be asked to stand up, walk 3 meters to a line on the floor at their usual speed, and return to a seated position in the chair. Participants will be allowed to use their usual gait aid used for indoors walking. The participant will be timed starting from when the instructor says “go”, and will stop when the participant sits again with their back against the back of the chair. The test will be repeated as a dual task test, using a secondary motor task by carry a cup of water. The time taken to complete the task is strongly correlated to level of mobility and activity of daily living performance [27].

ii. Lower body strength will be assessed using the Five Times Sit To Stand (FTSS) Test [20]. Participants will be asked to stand up and sit down from a 45 cm high chair five times as quickly as possible, with arms folded across their chest.

iii. Functional Reach test [28] is a dynamic test of standing balance in which the performance of reach ability in bilateral stance feet 10 cm apart will be evaluated. The participant will stand next to a wall with their feet 10 cm apart and dominant arm raised to 90 degrees. The initial distance of reach will be recorded. Then, participants will be asked to lean forward as far as they can without overbalancing and the distance of the additional reach will be recorded in centimetre (cm).

iv. Step Test [17] will be used to evaluate the speed of performing a dynamic repetitive single limb stance task (self-generated perturbation). The number of times the participant steps one foot fully on then off a 7.5 cm block step in 15 seconds will be recorded. Each foot will be tested separately. The worse side stepping (lowest score) will be used for analysis.

Laboratory assessment of balance related performance will be carried using Neurocom® Balance Master with long plate (Neurocom International, Inc.Clackamas, OR, USA). Practice trials will be provided on all tests to ensure the participant understands the test, prior to the actual measurement. All tests will be performed bare foot [29]. Five assessment procedures will be included:

I. Modified Clinical Test of Sensory Interaction of Balance (mCTSIB) -this test will measure the amount of sway under 4 sensory conditions (foot separation based on height, as detailed in user manual) [30] – eyes open and eyes closed on firm surface, and eyes open and eyes closed on a foam surface (medium density, 15 cm thick). Three trials are measured for each test, and the average sway from the three trials will be recorded. The composite sway velocity score (degrees/second), which was derived from the average of the sway velocity scores from the four sensory conditions, will be used in analyses.

II. Limits of stability (LOS) test (foot separation based on height, as detailed in user manual) [30] will be assessed to quantify the maximum distance and speed that a participant is able to intentionally displace their Centre of Gravity (COG), in shifting their weight towards eight targets positioned at 100% Limits of Stability. Maximum excursion (percentage of maximum limits of stability distance), which is the furthest distance of the center of gravity movement during testing, and directional control (percentage), which is a comparison of the amount of movement in the intended direction to the amount of extraneous movement, will be reported.

III. The Sit to Stand (STS) test will be used to quantify several movement characteristics as the participant rises from a seated to a standing position. The participant will sit on 41 cm high box placed centrally on the forceplate [30]. The participant’s feet are positioned equidistant from the center line. The participant will be instructed to hold steady until instructed to stand, and then to stand up quickly and to stand steady once upright. The sway exhibited during and immediately after performing the task (degrees/second) will be reported.

IV. The Walk Across (WA) test will be used to measure several characteristics of gait (gait velocity, step width, double support duration -% gait cycle) as the participant walks at comfortable pace across the long force plate [30].

V. The Step Quick Turn (SQT) test will be used to measure stability during 180 degree turn. The test involves the participant standing at one end of the long plate, and when instructed, to take one step with each leg (with a designated lead leg), then to turn 180 degrees quickly in the direction of lead leg, and return to the starting position, then to stand still [30]. Three trials will be repeated turning to the right, then three trials will be repeated turning to the left, and scores averaged for each direction of turning. Sway velocity (degrees/second) and turn time (s) will be reported for turning in each direction. For analysis, performance for the turn direction with worst performance (higher turn time or sway velocity) will be used.

Participants will be asked to wear an overhead harness as additional safety feature to prevent overbalancing during performing the modified CTSIB and Limits of Stability tests. The harness will be loose enough to allow normal balance reactions and upper body movement.

### Randomization

After the baseline assessment has been completed, participants will be randomly allocated to the home based exercise intervention or the control group (Figure 1). A computer generated random numbers table will be used, and a paper with the group number will be folded and concealed in numbered opaque envelopes by an independent staff member who is not part of the research team**.** Following the baseline assessment, the next consecutive envelope will be opened by a researcher not involved in the assessments, and the group allocation will be conveyed to the researcher implementing the intervention activities. To avoid any potential sampling contamination, any couples enrolled in the study will be randomized into the same group [31]. The participant will be informed as to which group they are allocated, and for those in the intervention group, an appointment will be made at the assessment laboratory for provision of the home exercise program.

### Intervention group activity

The intervention group will participate in a tailored (individualized) home based balance exercise program, provided by a trained researcher. This exercise program is based on the Otago program, a program shown to be effective in improving general balance in older people with mild balance dysfunction [29], but will include additional exercises selected to improve impairment of turning. The exercises selected for each participant in the intervention group will be based on assessment findings of areas of impairment. Two exercises of the 6–8 exercises prescribed will be selected to improve turning ability, while the other exercises will target other aspects of balance and mobility impairment based on the assessment findings. The selection of exercise and progression level will be tailored to the level of the participant ability. An exercise sheet will be provided by the researcher to each participant with illustrations and instructions detailing how to do the exercises, and dosage. The researcher will demonstrate each of the exercises and will ask the participant to perform the exercise just after the demonstration to ensure the participant understands how to do the exercise correctly. Participants will be provided with a written copy of the exercise program, and the researcher’s contact details if they need to discuss any aspect of the exercise program between visits. The home based exercise program is expected to take 20–30 minutes per session on average, including rests. Participants will be encouraged to continue the exercise program at least four times a week for the 16 weeks. The dosage is consistent with recommendations from a meta-analysis of exercise interventions in reducing falls [14]. In addition to the balance, strengthening and mobility related exercises, participants in the intervention group will also be provided with a graduated walking program, in which they will be asked to include a walking program 3 days/week, with gradually increasing distances.

Participants in the exercise intervention will return to the assessment laboratory for three occasions in the 16 week duration (one visit after 3, 6 and 9 weeks) to review the exercise program, modify exercises if required, and motivate the participant to persist with the exercises.

Adherence to exercise programs is an important determinant of outcome. Strategies to support adherence in this study include encouragement through phone calls between visits will be done by researcher who will prescribe and monitoring the exercise intervention. Participants will complete and return an exercise diary.

### Control group activity

Following the baseline assessment and randomization, the control group will be asked to continue with their usual activities.

### Follow up data collection

Intervention group participants will be asked to complete a daily log of their exercise participation, to be used to calculate exercise adherence. Both the intervention and control group will receive four phone calls interspersed over the 16 week period (weeks 4, 8, 12 and 16) by a researcher to obtain details regarding any falls that occurred between phone contacts.

### Safety

All assessments in this study will be carried out by trained researcher. The assessment procedures include a range of physical performance assessment tests, many of which are commonly used by physiotherapists involved in assessment and treatment of older people. The Neurocom® balance assessments are not commonly used by clinicians, but have been used safely in published research [29,32]. Participants will use a safety jacket and harness during several of the Neurocom® force platform balance assessments, which will ensure they will not overbalance during the assessment force platform tests. In other tests, they will be closely supervised by the research officer conducting the assessments. The individualized home based exercise is based on the Otago home based exercise programs that have been shown in a number of studies among at risk older people to be safely implemented [32,33]. The researcher implementing the exercise intervention will have training provided by an experienced physiotherapist (KH), and will use an exercise prescription algorithm developed for the study.

### Ethical considerations

The participant information sheet includes information on the purpose of the study, and potential risk, benefits and withdrawal procedures, which will be explained to the participants prior to seeking consent to participate. All information obtained through this study will remain confidential. Participant identifying data will only be used for the purpose of research and no individual identifiable information will be used in any types of publication. All data will be recorded on paper based forms, and will then be scanned to the electronic database. Electronic files will be stored on password protected computers, with access limited to members of the research team. All written files will be stored in locked filing cabinets in the research officer’s office, and will be archived and retained for five years after publication. At the end of this period, all paper based data will be shredded and the electronically data will be erased.

Ethics Committee approval has been received from Curtin University Research Ethics Committee (Approval No. FHEC PT231/2013, Approval Date: May 30,2013) and the Universiti Putra Malaysia Ethic Committee (Approval No. IG April Curtin (13) 04, Approval Date: May 10,2013). Permission to conduct research in Malaysia has been obtained from the Economic Planning Unit (EPU), Department of Prime Minister of Malaysia.

### Primary outcome measure

The primary outcome will be the Step Quick Turn test (Neurocom® Balance Master) (a laboratory measure) (amount of sway-degrees/s) and Timed Up and Go Test (a clinical measure). Other measures of balance and mobility performance, falls efficacy, and adherence to exercise program will be considered as secondary outcomes. While falls data is being collected, the study is not powered sufficiently to identify a significant difference in falls between the two groups.

### Statistical analysis

Baseline and post intervention measures will be reported as means (m) ± standard deviations (SD). Data will be analysed on an intention to treat basis. Change in the selected primary outcome measure and secondary outcomes will be evaluated using two way Repeated Measures ANOVA to determine group main effect, time main effect, and interaction effect between group and time. Level of significance will be set at P < 0.05. Participants in the exercise intervention group will be grouped according to high adherence (> median adherence for the full group) and low adherence (≤ median adherence level). Repeated measures ANOVA will be used to determine whether there is a difference in change in the primary and secondary balance measures between the high adherence and low adherence group.

### Sample size

Power analysis was performed for the primary outcome measure (Step Quick Turn test on the Neurocom® Balance Master), using the mean score for the Step Quick Turn test (sway-degrees/s) results from a previous exercise intervention trial of older people with mild balance impairments [34]. It was estimated that 34 participants per group (68 participants in total) would be required to have at least 80% power to detect a 15% improvement in the Step Quick Turn test using the turn sway measure on the Neurocom Balance Master long-plate (degrees/s), (assuming 50% standard deviation improvement with the intervention [sd = 10.2, effect size = 0.5] at P < 0.05 (two-tailed). This calculation includes allowance for an anticipated 20% dropout rate. This sample size should also be adequate for other secondary outcome measures including the Limits of Stability test (Neurocom® Balance Master), the Step Test, and Functional Reach Test.

### Missing data

Missing data (due to loss to follow up) will be imputed using the last value carried forwards method [35].

## Discussion

Given the importance of balance for independence and safety for older people, and the negative effect of age and health problems on balance ability, exercise has been a widely investigated intervention to improve balance and reduce falls risk [36,37]. A Cochrane review reported that multi-component group exercise, typically including resistance and balance training, reduced the rate of falls by 22% and falls risk by 17% in adults aged 60 years and over [38]. Howe and colleagues [39] analysed results from 34 studies of exercise interventions aiming to improve balance. The review had a total of 2883 participants and a variety of exercise interventions, including walking, functional exercises, muscle strengthening, and combined exercise types, that were found to significantly improve balance. A number of balance intervention studies have focused on task specific exercises and every day activities such as getting in and out of a chair, or stepping up and down from one level to another [40-43]. However, despite the importance of turning ability, and involvement of turning in the circumstances of falls, there has been very little research focus on identifying turning impairments in older people at risk of falls, or in evaluating interventions to improve turning performance. This study will address this important gap in the current literature.

Another important aspect of this study’s method is that it involves recruitment of participants who remain relatively mobile and do not have high levels of falls risk. As such, from a health promotion and intervention perspective, if the intervention is shown to be effective, it has potential to be applied before balance dysfunction and falls become too advanced. In addition, improving functional performance at this relatively mild stage of falls risk will assist to sustain and maintain the functional capability and mobility performance of community dwelling older adults.

Finally, there has been little falls prevention intervention programs conducted in Malaysia, and there is some evidence to suggest that interventions effective in Western countries (where the majority of falls prevention research has been carried out) may not always be translatable to Asian countries [44]. Differences in socioeconomic, cultural, behavioral and environmental factors may all impact upon the likely success of interventions between different countries. This study will provide some valuable local data regarding risk factors for falls among older community dwelling Malaysians, and the acceptability of this type of home exercise program in the Malaysian context. This study is proposed to provide a basis for planning future fall prevention interventions of relevance to specific identified falls risk in the Malaysian population.

There are several elements of our proposed method that have been adopted to accommodate the limited timelines and resourcing available for the project. There is some debate in the research literature relating to the accuracy of self-report measures of physical activity, and that accelerometers provide a more objective and valid measure [45]. The Human Activity Profile self-report measure selected for use in this project has been extensively used in research, and has been shown to be reliable and valid [46]. The duration of follow up for this study was limited to immediately following the four months intervention, instead of the recommended 12 month follow up for fall prevention studies [47]. Falls data is being collected as a secondary outcome measure to inform future study power calculations, however the study is not powered to detect a significant difference on the falls measure. These limitations are not expected to impact on the overall project aim of evaluating the effectiveness of this exercise program in improving turning performance.

## Competing interests

The authors declare that they have no competing interests.

## Authors’ contributions

AA and KH were involved in design, preparing and revising the manuscript; TAH participated in the study design, and TAH and MH critically reviewed the manuscript. All authors read and approved the final manuscript.

## Pre-publication history

The pre-publication history for this paper can be accessed here:

http://www.biomedcentral.com/1471-2318/14/100/prepub
